# A structured overview of trends and technologies used in dynamic hand orthoses

**DOI:** 10.1186/s12984-016-0168-z

**Published:** 2016-06-29

**Authors:** Ronald A. Bos, Claudia J.W. Haarman, Teun Stortelder, Kostas Nizamis, Just L. Herder, Arno H.A. Stienen, Dick H. Plettenburg

**Affiliations:** Department of Biomechanical Engineering, Delft University of Technology, Mekelweg 2, 2628 CD Delft The Netherlands; Department of Biomechanical Engineering, University of Twente, Drienerlolaan 5, 7522 NB Enschede The Netherlands; Department of Precision and Microsystems Engineering, Delft University of Technology, Mekelweg 2, 2628 CD Delft The Netherlands; Department of Physical Therapy and Human Movement Sciences, Northwestern University, 645 N. Michigan Ave. Suite 1100, Chicago, 60611 IL USA

**Keywords:** Hand impairments, Orthosis, Exoskeleton, Rehabilitation robot, Assistive device

## Abstract

The development of dynamic hand orthoses is a fast-growing field of research and has resulted in many different devices. A large and diverse solution space is formed by the various mechatronic components which are used in these devices. They are the result of making complex design choices within the constraints imposed by the application, the environment and the patient’s individual needs. Several review studies exist that cover the details of specific disciplines which play a part in the developmental cycle. However, a general collection of all endeavors around the world and a structured overview of the solution space which integrates these disciplines is missing. In this study, a total of 165 individual dynamic hand orthoses were collected and their mechatronic components were categorized into a framework with a signal, energy and mechanical domain. Its hierarchical structure allows it to reach out towards the different disciplines while connecting them with common properties. Additionally, available arguments behind design choices were collected and related to the trends in the solution space. As a result, a comprehensive overview of the used mechatronic components in dynamic hand orthoses is presented.

**Electronic supplementary material:**

The online version of this article (doi:10.1186/s12984-016-0168-z) contains supplementary material, which is available to authorized users.

## Background

Human hands are complex and versatile instruments. They play an essential role in the interaction between a person and the environment. Many people suffer from hand impairments like spasticity, lack of control or muscle weakness, which may be due to the consequences of stroke, paralysis, injuries or muscular diseases. Such impairments may limit an individual’s independence in performing activities of daily living (ADL) and the ability to socially interact (e.g. non-verbal communication). Devices like hand exoskeletons, rehabilitation robots and assistive devices, here collectively termed as dynamic hand orthoses, aim to overcome these limitations. Their development is a fast-growing field of research and has already resulted in a large variety of devices [1–4].

Each individual has different demands for a dynamic hand orthoses. Some patients benefit from rehabilitation therapy (e.g. stroke patients [5]) while others would more likely benefit from daily assistance (e.g. Duchenne Muscular Dystrophy [6]). The resulting diversity between the different devices can be illustrated by the elaborate overviews on robotic devices [4], training modalities [3] and intention detection systems [7] they use. Clearly, there are many mechatronic components to choose from and are often the result of making particular design choices within the imposed design constraints. However, not everybody has the resources (i.e. time, accessibility) to investigate all possible design choices within these constraints. Moreover, not always are design choices reported in literature and are therefore hard to retrieve. The full potential of learning from each other’s endeavors is therefore not yet fully exploited, leaving several questions in this field of research unanswered. For example, there is the discussion whether pneumatic or electric actuation is better for some applications.

The goal of this study is to collect a high quantity of dynamic hand orthoses and extract the mechatronic components which are used. Their collective properties are analyzed by using a framework which uses a generic categorization applicable for any mechatronic system: a signal domain (e.g. controllers, sensors), energy domain (e.g. energy sources, actuators) and mechanical domain (e.g. cables, linkages). Additionally, feasible technologies from other, but similar, disciplines are included (e.g. prosthetics, haptics). Trends are then visualized using bar charts and compared to available arguments behind design choices. This not only includes arguments from often-cited success-stories, but also from small-scale projects. Referring to the case of using pneumatic or electric actuation, this approach can answer how often each method is used and what arguments are reported, which may help in scoping further research and making a well-considered choice.

This paper is structured in different sections. The “Scope” section describes the boundaries and limitations of this study and Framework introduces the basis of the framework structure that is proposed. The “Results” section describes the quantitative results which illustrate the trends. How this relates to the functionality of the components, is discussed and summarized in the “Discussion” and “Conclusion” section, respectively.

## Scope

### Search strategy

The used terminology often varies between studies due to different backgrounds or field of application. For example, the term ‘exoskeleton’ has been presented as a type of rehabilitation robot [4] or, conversely, as a device that is not used for limb pathologies but to augment the strength of able-bodied people [8]. In this study, following the example from [9] and in conformity with ISO 8549-1:1989 [10], the term ‘orthosis’ is used to cover the full range of applications. The added term ‘dynamic’ then provides a scope towards devices that facilitate movement.

**Fig. 1:**
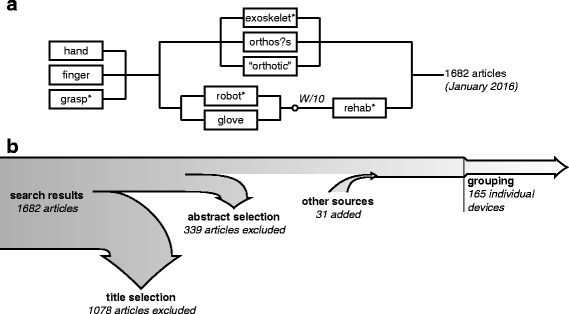
Search query and selection procedure. **a** A visualization of the search query is shown which resulted in 1682 articles. Here, connections in series represent ’AND’ and proximity (’W/10’) operators, those in parallel represent ’OR’ operators. **b** The results underwent title/abstract selection based on inclusion criteria and more sources were added through references/citations. Finally, articles were grouped in order to extract the individual devices

In order to collect a large quantity of dynamic hand orthoses, sources of literature were searched in Scopus, where a set of keywords was used to search in titles and abstracts. A visual representation of the search query and selection procedure can be seen in Fig. 1.

Boolean operators and wildcard symbols were used to include alternative spellings and synonyms. The used search query was (hand OR finger OR grasp*) AND ((rehab* W/10 robot* OR glove) OR (exoskelet* OR orthos?s OR “orthotic”)). The inclusion criteria were defined as regular articles in the English language which presented a dynamic orthosis, supporting at least a finger joint. Using standardized terminology from ISO 8549-3:1989 [11], this includes the finger orthosis (FO), hand orthosis (HdO) and wrist-hand-finger orthosis (WHFO). The wrist-hand orthosis (WHO) was not included, as it stems from the deprecated term wrist orthosis (WO) [11] and therefore does not necessarily support a finger joint. Whenever a combined arm and hand support system was presented, e.g. a shoulder-elbow-wrist-hand orthosis (SEWHO), only the hand and wrist module was included. Based on the inclusion criteria, the search results underwent a title and abstract selection. Additional sources were added from relevant citations and references, as well as other possibly linked publications from the same author(s)/institution(s). Ultimately, this resulted in a total of 296 articles, describing 165 unique devices. Other supplementary sources of information used in this study include websites/brochures for commercial devices, key review studies, standards and articles describing fundamentals on specific topics.

Year of publication was considered to cover the temporal aspect of trends and technology. Devices were placed into groups of before 2006, 2006–2010 and 2011–2015, where a device’s year was defined by the most recent publication in which change to the design is reported.

### Applications

As a preliminary classification, the dynamic hand orthoses were split up into different applications. These can be both medical and non-medical. Medical applications focus on enhancing or recovering hand function for a wide range of patients with disabilities in the hand. Non-medical applications, on the other hand, focus on haptic interfaces or providing additional strength for more demanding tasks. In many cases, a device’s application was explicitly stated in available literature, whereas in other cases it needed to be derived from the imposed design constraints. In the latter case, the most restrictive constraints were used as distinguishing features (e.g. strict constraints on portability can indicate home use). The different applications which were used are described below.

A research tool is often used for making accurate measurements, investigating the fundamental working principle and properties of the hand [12]. Additionally, they can be used to simulate different treatments and analyze the ideal strategies for other applications [13]. Emphasis is mostly put on accuracy and reliability, rather than size and ease of use.

A clinical tool can be used for diagnostic purposes, but are mostly used for robot-assisted rehabilitation at the clinic with reduced active workload for the professional caregiver [5, 14–16].

A home rehabilitation tool can be similar to a clinical tool, but does not require personal supervision and poses more strict design constraints regarding to its size, portability and ease of use. Examples are systems that use continuous passive motion (CPM) and/or virtual reality (VR) environments, in which fun and gaming are critical aspects for increasing patient motivation [16, 17]. In most cases, progress is remotely or occasionally monitored by a clinician, allowing for personalized rehabilitation programs and the ease of staying at home. This is an increasingly popular field in rehabilitation devices, as it ideally reduces time in the clinic and maximizes hours of physical therapy [5].

A daily assistive tool is intended to assist during ADL. These types of devices are meant to be used for several hours a day without supervision from a caregiver. They are more invasive to a person’s daily routine and, similar to prosthetics [18], the comfort, cosmesis and control presumably become key factors. They differ from home rehabilitation tools as they aim to assist in task execution, rather than to perform physical therapy. Sometimes physical therapy can be offered through assistance [19], in which case the daily assistance imposes the most restrictive design constraints.

A haptic device is originally a non-medical device and is used as a master hand. They interact with a VR environment or perform teleoperation while providing the user with haptic feedback. Due to similar design constraints, haptic devices become comparable with medical applications and are sometimes reported to be able to perform both (e.g. [20, 21]).

Lastly, Extra-Vehicular Activity (EVA) gloves for astronauts are included as a non-medical application. Their intended function is to compensate the high stiffness of an astronaut’s gloves during activities that require a space-suit. Similar to haptic devices, these devices are included due to comparable design constraints (e.g. [22, 23]).

## Framework

### Structure

**Fig. 2:**
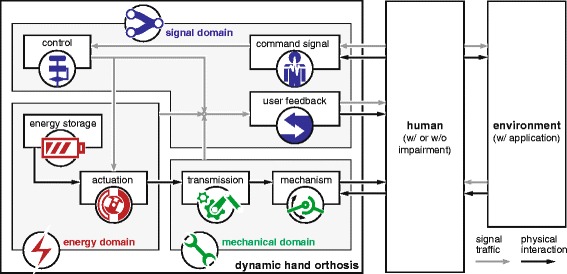
Basic interactions for a dynamic hand orthosis. The device consists of several components which can be categorized into the signal, energy and mechanical domain. Gray arrows represent signal traffic, which can be made of visual or auditory stimuli, as well as electrical currents used for artificial control or the nervous system. Black arrows indicate physical interactions in the form of forces and motions. The human interacts with the device through its mechanism, but additional interactions can be provided through the command signal or user feedback

In order to collectively analyze a large quantity of dynamic hand orthoses, a framework was constructed which uses the concept of tree diagrams. Firstly, the basic components of a dynamic hand orthosis were identified. Their relations are illustrated in Fig. 2, along with the interactions with the human and environment. Also shown in this figure, is a division of these components into three different domains:

- **signal** domain (controller, command signal, user feedback): determines the training modalities, how the human can control the device and how the human is informed about the device’s status;
- **energy** domain (energy storage, actuation): determines the source of energy and the conversion into mechanical work that is applied through the system;
- **mechanical** domain (transmission, mechanism): determines how mechanical work is transported and how the different joints are supported.

**Fig. 3:**
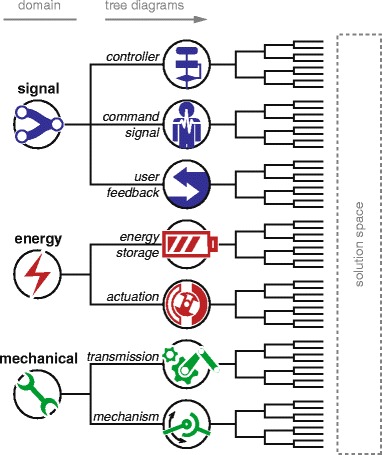
Conceptual framework. Several tree diagrams are categorized into the signal, energy and mechanical domain. Towards the right side, branches lead to the solution space in increasing level of detail

These domains were chosen such that they are all-inclusive and describe a generalized mechatronic system that interacts with a human. Starting from these general domains, tree diagrams were defined which describe the mechatronic components that make up the solution space. See Fig. 3 for a schematic. At each branching point, the level of detail increases. This method was chosen as it visualizes possible design choices at several levels of detail and categorizes them among three separable domains.

### Characteristics & limitations

The proposed framework was used as a subjective tool from which objective observations could be made. This is because there are multiple ways of defining the branching points, as long as the divisions are as all-inclusive as possible to accommodate all possible solutions. Moreover, it was constructed in order to discuss components and trends as a whole, rather than scoping down into full detail which is already covered in other useful reviews and classifications [3, 7, 24–26]. Existing relevant methods and terminology from these studies were used as much as possible, such that their definitions are covered in their respective sources.

The process of categorization involved investigating the available literature for each device and checking which ends of the tree branches were used. By counting all checked occurrences, the trends for each tree branch could be seen in terms of numbers grouped by year ranges. It is important to note that these numbers indicate a rate of popularity and does not always correlate to functionality, which is treated in the Discussion section. High numbers could arise because something is successful, easily accessible or common practice. Low numbers, on the other hand, could indicate that the respective solution is still experimental, not easily accessible, not well-known or it simply does not work for a given application.

**Fig. 4:**
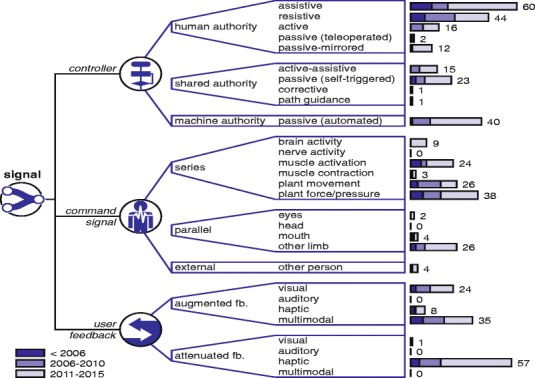
Signal domain. Tree diagrams within the signal domain and their number of occurrences in found devices, grouped by year ranges

**Fig. 5:**
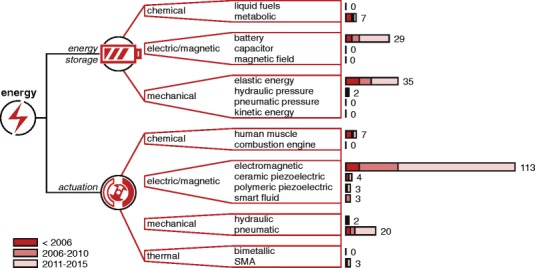
Energy domain. Tree diagrams within the energy domain and their number of occurrences in found devices, grouped by year ranges

**Fig. 6:**
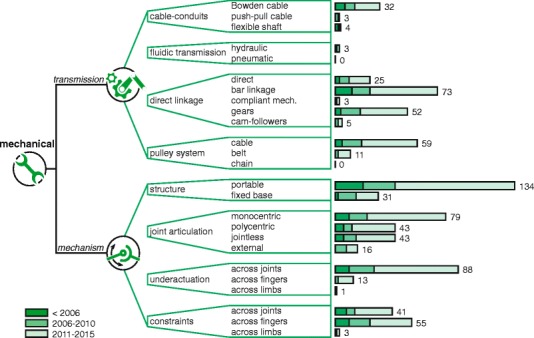
Mechanical domain. Tree diagrams within the mechanical domain and their number of occurrences in found devices, grouped by year ranges

A visualization of the completed framework can be seen in Figs. 4, 5 and 6 as part of the “Results” section. Embedded in this framework is a set of terms, which are discussed below per domain.

#### Signal

The first tree diagram within this domain encompasses the training modalities from [3] employed by the controller, subdivided according to who has authority over the device’s movement [27]. The passive modality appears three times due to this additional subdivision. Automated passive training (machine authority) most resembles the traditional passive training modality. From a patient’s perspective, self-triggered passive training (shared authority) can be considered to invoke different cognitive processes and—depending on the trigger—approaches the situation of an active-assistive modality. From the device’s perspective, teleoperated passive training (human authority) implies different lower level control strategies. A second tree diagram covers the command signal required to activate the device, similar to [7]. The third tree diagram describes the modes of feedback which are available to the user, using principles from motor learning [24]. Here, standard physiological feedback is assumed and changes due the orthosis by augmentation or attenuation were considered.

#### Energy

Within the energy domain, the tree diagrams incorporate types of energy storage and actuation. The diagrams have a similar structure and are subdivided according to feasible types of energy and stimulus from [25] and [26]. Methods of energy storage were scoped towards portable solutions. Nuclear, wind and solar energy were considered infeasible, as well as using thermal energy for energy storage.

#### Mechanical

For an all-inclusive incorporation of components in the mechanical domain, one can refer to Reuleaux’s classification of kinematic pairs from 1876, largely available as a digital library from the Cornell University [28]. Instead, to make the framework more compact, a more crude categorization is proposed in terms of principles encountered in dynamic hand orthoses. Hence, the first tree diagram includes transmission components which are used to transfer mechanical energy, whereas the second tree diagram describes the mechanism by its shape (i.e. structure), how the anatomical joints are supported (i.e. joint articulation) and which couplings are added to simplify the mechanism (i.e. underactuation and constraints). More specifically for joint articulation, the axis of rotation is monocentric or polycentric according to ISO 13404:2007 [29]. Jointless and external methods of articulation were added to also encompass glove and end-effector types of devices, respectively.

## Results

A total of 165 different dynamic hand orthoses were found, of which 109 cases presented changes most recently published between 2011 and 2015. A list of all devices is divided according to application and is shown in Tables 1, 2, 3, 4, 5 and 6. These tables contain relevant references and additional descriptive information per device. See Additional file 1 for more detailed information on these devices and their individual categorization.

**Table 1:** Overview of included dynamic hand orthoses classified as research tool

Name/ID	Country	Year range^a^	ISO abbr.	Reported function	Actuator DOF^b^	Wrist support^b^
MR_CHIROD v.2 [113–115]	USA	2005–2008	HdO	post-stroke measurement	1	N/A
FingerBot [48]	USA	2010	FO	post-stroke measurement	3	N/A
ATX [116]	USA	2011	FO	post-stroke measurement	5	N/A
Fiorilla [12, 117]	Italy	2009–2011	FO	normal measurement	2	Limited (PS) Locked (FE, RUD)
Ramos [118, 119]	Germany	2009–2012	WHFO	post-stroke therapy	4	Locked (PS, FE, RUD)
Tang [120–123]	Japan	2011–2013	FO	post-stroke measurement/therapy	1	Limited (FE)
CAFE [13, 124, 125]	USA	2007–2014	FO	post-stroke measurement	6	Locked (FE, RUD)
Kim 2 [126]	South Korea	2015	WHFO	general measurement/therapy	1	Limited (PS, FE, RUD)
Lee 2 [127]	South Korea	2015	HdO	post-stroke measurement	5	N/A

^a^Year ranges are determined by the year span between found literature sources and my differ from the actual time of development

^b^Actuator DOF = number of individually controlled actuators (zero means fully passive)

^c^Wrist can be assisted, resisted, limited or locked [29] in pronation/supination (PS), flexion/extension (FE) and radial/ulnar deviation (RUD)

**Table 2:** Overview of included dynamic hand orthoses classified as clinical tool

Name/ID	Country	Year range^a^	ISO abbr.	Reported function	Actuator DOF^b^	Wrist support^b^
HWARD [30]	USA	2005	WHFO	post-stroke therapy	2 (+1 wrist)	Locked (PS, RUD) Assisted (FE)
HIFE [20]	Slovenia	2006	FO	general physical therapy	2	Locked (PS, FE, RUD)
Gentle/G hand device [49]	UK	2007	WHFO	post-stroke therapy	3	Locked (FE, RUD)
InMotion Hand Robot [89, 128, 129]	USA	1991–2007	HdO	post-stroke therapy	1	N/A
CPM/CAM [130]	Canada	2008	HdO	general CPM/CAM	2	N/A
Fu [90, 131]	China	2008	FO	general CPM	2	N/A
ADLER FES grasp glove [132, 133]	USA	2007–2009	HdO	post-stroke therapy	Not clear	Not clear
IntelliArm hand module [134, 135]	USA	2008–2009	WHFO	post-stroke measurement/therapy	1 (+2 wrist)	Assisted (PS, FE) Locked (RUD)
Sun [72, 136]	China	2006–2009	WHFO	post-stroke therapy	2	Limited (PS, FE, RUD)
Wang [137–141]	China	2009–2011	FO	general physical therapy	4	Limited (FE, RUD)
Yamaura [142]	Japan	2009	FO	general physical therapy	2	N/A
HenRiE grasp module [143–145]	Slovenia	2008–2010	WHFO	post-stroke therapy	0	Locked (FE, RUD)
HEXORR [64]	USA	2010	WHFO	post-stroke therapy	2	Locked (PS, FE, RUD)
PneuGlove [146–148]	USA	2006–2010	HdO	post-stroke therapy	5	N/A
Unluhisarcikli [149]	USA	2008–2010	WHFO	post-stroke therapy	2 (+1 wrist)	Assisted (PS)
ExoHand [21]	Germany	2012	WHFO	tele-operation, post-stroke therapy	8	Limited (FE, RUD)
iHandRehab [91, 137, 150]	China	2009–2012	HdO	general physical therapy	8	N/A
Kim 1 [151]	South Korea	2013	WHFO	post-stroke therapy	10	Locked (PS) Limited (FE, RUD)
Sooraj [152]	India	2013	WHFO	general physical therapy	5	Locked (PS, FE, RUD)
Amadeo [153–155]	Austria	2010–2014	WHFO	general measurement/therapy	5	Locked (PS, FE, RUD)
AMES hand module [53, 54, 156, 157]	USA	2009–2014	WHFO	post-stroke therapy	1 (+1 wrist)	Locked (PS, RUD) Assisted (FE)
AssistOn-Finger [109, 158]	Turkey	2009–2014	FO	tendon injury treatment	1	Locked (FE, RUD)
Bi [159–161]	China	2011–2014	WHFO	post-stroke therapy	5	Locked (FE, RUD)
Chan [162]	Malaysia	2014	HdO	post-stroke therapy, general assistance	3	N/A
FINGER [52, 163, 164]	USA	2011–2014	FO	post-stroke therapy	1	Locked (PS, FE, RUD)
HIT-Glove [165–168]	China	2010–2014	FO	post-stroke therapy	6	N/A
Kawasaki [44, 169–172]	Japan	2004–2014	WHFO	post-stroke therapy	16 (+ 2 wrist)	Assisted (PS, FE) Locked (RUD)
King [47, 173, 174]	USA	2009–2014	HdO	post-stroke therapy	7	N/A
PMHand [175]	UK	2014	HdO	post-stroke therapy	1	N/A
ReachMAN2 [106, 176, 177]	UK	2009–2014	WHFO	post-stroke therapy	1 (+1 wrist)	Assisted (PS) Locked (FE, RUD)
Reha-Digit [178–180]	Germany	2008–2014	HdO	general CPM	1	Limited (PS, FE, RUD)
Ushiba [181]	Japan	2014	WHFO	post-stroke therapy	1	Locked (FE, RUD)
IHRG [182–187]	Romania	2013–2015	HdO	post-stroke therapy	4	N/A
READAPT [188–192]	USA	2008–2015	WHFO	post-stroke measurement/therapy	8 (+3 wrist)	Assisted (PS, FE, RUD)

^a^Year ranges are determined by the year span between found literature sources and my differ from the actual time of development

^b^2Actuator DOF = number of individually controlled actuators (zero means fully passive)

^c^Wrist can be assisted, resisted, limited or locked [29] in pronation/supination (PS), flexion/extension (FE) and radial/ulnar deviation (RUD)

**Table 3:** Overview of included dynamic hand orthoses classified as home rehabilitation tool

Name/ID	Country	Year range^a^	ISO abbr.	Reported function	Actuator DOF^b^	Wrist support^b^
Sarakoglou [50]	UK	2004	HdO	general physical therapy	7	N/A
Luo [193, 194]	USA	2005	HdO	post-stroke therapy	1	N/A
Mulas [195]	Italy	2005	WHFO	general physical therapy	2	Limited (PS, FE, RUD)
Haptic Knob [31, 196]	Singapore	2007	WHFO	post-stroke therapy	1 (+1 wrist)	Assisted (PS) Limited (FE, RUD)
MRAGES [197]	USA	2007	HdO	general physical therapy	5	N/A
Wege [37, 87, 198–200]	Germany	2005–2007	HdO	general physical therapy	20	N/A
Carpi [201]	Italy	2008	WHFO	general impairment compensation	1	Locked (FE, RUD)
HandCARE [202]	Singapore	2008	HdO	post-stroke therapy	1	Limited (PS, FE, RUD)
Chen [203]	China	2009	WHFO	post-stroke therapy	5	Locked (FE, FE, RUD)
HIRO III [204]	Japan	2010	HdO	general physical therapy	15	N/A
Mohamaddan [205]	Malaysia	2010	HdO	post-stroke therapy	2	N/A
NeReBot hand add-on [60, 206]	Italy	2009–2010	WHFO	post-stroke therapy	1	Locked (PS, FE, RUD)
Burton [207, 208]	UK	2011–2012	WHFO	post-stroke therapy	6	Limited (FE, RUD)
J-Glove [40, 209]	USA	2009–2011	WHFO	post-stroke therapy	1	Locked (FE, RUD)
PRoGS [210]	Singapore	2010–2011	WHFO	post-stroke therapy	5	N/A
SaeboFlex [211, 212]	USA	2011	WHFO	post-stroke therapy, hypertonia compensation	0	Locked (FE, RUD)
Tzemanaki [213]	UK	2011	HdO	general therapy	5	N/A
DULEX-II [45, 214]	South Korea	2009–2012	WHFO	post-stroke therapy	2 (+ 1 wrist)	Assisted (FE) Locked (RUD)
ExoFlex [215]	USA	2012	HdO	general therapy	4	N/A
HANDEXOS [98, 216, 217]	Italy	2009–2012	FO	post-stroke therapy	1	N/A
JACE H440 Hand CPM [218]	USA	2012	WHFO	general physical therapy	1	Locked (PS, FE, RUD)
Kazemi [219]	Canada	2012	WHFO	post-stroke measurement/therapy	1 (+1 wrist)	Assisted (PS)
Naidu [220, 221]	South Africa	2011–2012	WHFO	post-stroke therapy	2 (+1 wrist)	Assisted (PS) Locked (FE, RUD)
Polotto [222]	Canada	2012	FO	post-stroke therapy/assistance	4	N/A
WaveFlex Hand CPM [223–225]	USA	1997–2012	WHFO	general physical therapy	1	Locked (FE, RUD)
Wu [71, 75, 226–229]	China	2008–2012	WHFO	post-stroke therapy	2	Limited (PS) Locked (FE, RUD)
CAFEx [230]	Malaysia	2013	HdO	post-stroke therapy	1	N/A
Gloreha Lite [231, 232]	Italy	2013	HdO	general physical therapy	5	N/A
Hand of Hope [105, 233–236]	China	2010–2013	HdO	post-stroke therapy	5	N/A
mRes [95]	Germany	2013	HdO	post-stroke therapy	4	N/A
Orlando [237, 238]	India	2010–2013	FO	post-stroke therpapy	3	N/A
Rahman [239, 240]	Australia	2012–2013	WHFO	post-stroke therapy	5	N/A
Shafi [241]	Pakistan	2013	HdO	general physical therapy	4	N/A
Song [242]	Taiwan	2013	HdO	post-stroke therapy/assistance	3	Limited (FE)
UoA hand exoskeleton [61, 74]	Australia	2012–2013	WHFO	post-stroke therapy	11	Limited (PS, FE, RUD)
BiomHED [97, 243, 244]	USA	2014	WHFO	post-stroke therapy	7	Limited (PS) Locked (FE, RUD)
Coffey [245]	Ireland	2014	WHFO	post-stroke therapy	1	Limited (PS, RUD) Assisted (FE)
Guo [246]	China	2014	FO	post-stroke therapy	1	N/A
HEXOSYS-I [86, 247, 248]	Italy	2010–2014	HdO	general physical therapy	2	N/A
IOTA [249]	USA	2014	WHFO	pediatric rehabilitation	2	N/A
Maestra [250, 251]	France	2014	WHFO	general physical therapy	1	Assisted (PS, FE, RUD)
Maestra Portable [250, 251]	France	2014	WHFO	general physical therapy	1	Locked (FE, RUD)
PAFEx [38, 252]	Japan	2009–2014	HdO	post-stroke therapy	3	N/A
Pu [253, 254]	Taiwan	2014–2015	WHFO	general physical therapy	4	Locked (FE, RUD)
ReHand-II [255, 256]	China	2014	HdO	post-stroke therapy	2	N/A
ReHapticKnob [257–259]	Switzerland	2011–2014	WHFO	post-stroke measurement/therapy	1 (+1 wrist)	Assisted (PS)
SPO [32, 260, 261]	Netherlands	2013–2014	WHFO	post-stroke therapy	0	Resisted (F), Assisted (E) Locked (RUD)
Tang 2 [81, 262]	China	2013–2014	HdO	general physical therapy	10	N/A
ULERD hand module [263, 264]	China	2013–2014	WHFO	post-stroke therapy	1 (+2 wrist)	Assisted (PS, FE) Locked (RUD)
Ab Patar [265, 266]	Japan	2015	HdO	post-stroke therapy	3	N/A
HEXOSYS-II [267–270]	Italy	2010–2015	WHFO	general physical therapy	5	Limited (FE, RUD)
HX [96, 271–274]	Italy	2013–2015	WHFO	general physical therapy	2	Locked (RUD)
NESS H200 [275, 276]	USA	1996–2015	WHFO	general physical therapy	Not clear	Not clear
Ramirez [277]	Mexico	2015	WHFO	general physical therapy	6	Limited (PS) Locked (FE, RUD)
Richards [278]	UK	2015	HdO	post-stroke rehabilitation	2 (+1 palm)	N/A
SAO-i3 [279, 280]	Netherlands	2014–2015	WHFO	post-stroke therapy	1	Assisted (FE, RUD)

^a^Year ranges are determined by the year span between found literature sources and my differ from the actual time of development

^b^Actuator DOF = number of individually controlled actuators (zero means fully passive)

^c^Wrist can be assisted, resisted, limited or locked [29] in pronation/supination (PS), flexion/extension (FE) and radial/ulnar deviation (RUD)

**Table 4:** Overview of included dynamic hand orthoses classified as daily assistive tool

Name/ID	Country	Year range^a^	ISO abbr.	Reported function	Actuator DOF^b^	Wrist support^b^
Hardiman project [281–283]	USA	1967–1971	WHFO	power assistance	2 (+2 wrist)	Assisted (PS, FE) Locked (RUD)
Hamonet [284]	France	1974	HdO	tetraplegic assistance	1	N/A
K U finger splint S-type [285]	Japan	1978	WHFO	general impairment compensation	0	Limited (FE, RUD)
K U finger splint W-type [285]	Japan	1978	WHFO	general impairment compensation	0	Limited (FE, RUD)
WDFHO [102, 286, 287]	USA	1978–2013	WHFO	tetraplegic assistance	1	Assisted (FE), Locked (RUD)
Dollfus [288]	France	1984	HdO	tetraplegic assistance	1	N/A
Benjuya [51]	USA	1990	HdO	tetraplegic assistance	1	N/A
Slack [289]	Canada	1992	WHFO	tetraplegic assistance	1	Limited (PS, FE, RUD)
Brown [290]	USA	1993	HdO	tetraplegic assistance	5	N/A
SMART WHO [80]	Canada	1993	WHFO	tetraplegic assistance	1	Limited (FE), Locked (RUD)
DiCicco [291]	USA	2004	WHFO	tetraplegic assistance	2	Limited (FE, RUD)
Watanabe [55, 292]	Japan	2005–2007	WHFO	arthritis assistance	1	Locked (FE, RUD)
Alutei [293]	Romania	2009	WHFO	general assistance	1 (+1 wrist)	Assisted (PS) Locked (FE, RUD)
Moromugi 1 [173]	Japan	2009	HdO	general assistance	7	N/A
Exo-Finger [46]	Japan	2010	HdO	post-stroke assistance	1	N/A
Moromugi 2 [294]	Japan	2010	HdO	tetraplegic assistance	1	Locked (RUD)
Tadano [73]	Japan	2010	HdO	power assistance	10	N/A
HandSOME [62]	USA	2011	WHFO	post-stroke impairment compensation	0	Locked (FE, RUD)
PowerGrip [295]	USA	2011	WHFO	general assistance	1	Locked (FE, RUD)
Toya [296]	Japan	2011	HdO	general assistance	4	N/A
Baqapuri [297]	Pakistan	2012	WHFO	tetraplegic assistance	4	Limited (PS, FE, RUD)
SEM Glove [94]	Sweden	2012	HdO	general assistance	3	N/A
Arata [63]	Japan	2013	HdO	general therapy/assistance	1	Limited (FE, RUD)
KULEX grasping module [298–300]	South Korea	2012–2013	WHFO	general assistance	1 (+3 wrist)	Assisted (PS, FE, RUD)
Lambercy [301]	Switzerland	2013	FO	post-stroke therapy/assistance	1	N/A
Moromugi 3 [302]	Japan	2013	HdO	tetraplegic assistance	3	N/A
MUNDUS hand orthosis [36]	Italy	2013	HdO	tetraplegic assistance	1	N/A
Zheng [82]	China	2013	HdO	general assistance	Not clear	Not clear
Aw [83]	Australia	2014	HdO	general assistance	14	N/A
Kudo [303]	Japan	2014	HdO	tetraplegic assistance	1	N/A
Lee 1 [84, 304]	South Korea	2012–2014	HdO	general assistance	5	N/A
Nishad [305]	India	2014	HdO	general therapy/assistance	8	Limited (FE, RUD)
OFX [58, 306, 307]	South Korea	2013–2014	WHFO	general assistance	1	Locked (FE, RUD)
Puzo [308]	USA	2014	HdO	general therapy/assistance	5	N/A
SaeboGlove [212]	USA	2014	WHFO	general impairment compensation	0	Locked (FE, RUD)
Sasaki [39, 41, 309]	Japan	2004–2014	HdO	general assistance	5	N/A
BRAVO Hand Exoskeleton [310–312]	Italy	2011–2015	HdO	post-stroke therapy/assistance	2	N/A
Conti [313]	Italy	2015	HdO	general assistance	4	N/A
Cui [314]	Australia	2015	HdO	general assistance	5	N/A
Delph II [99, 315]	USA	2013–2015	HdO	post-stroke therapy/assistance	5	N/A
ExoGlove [316–319]	Singapore	2015	HdO	general therapy/assistance	1	N/A
Gasser [320]	USA	2015	HdO	post-stroke assistance	2	N/A
Hasegawa [321–326]	Japan	2008–2015	WHFO	power assistance	8 (+ 4 wrist)	Assisted (PS, FE)
OHAE [92, 327–330]	USA	2009–2015	WHFO	general assistance	3	Limited (FE, RUD)
Polygerinos [19, 331, 332]	USA	2013–2015	HdO	general therapy/assistance	4	N/A
SNU Exo-Glove[85, 93, 333–335]	South Korea	2011–2015	WHFO	general therapy/assistance	3	N/A

^a^Year ranges are determined by the year span between found literature sources and my differ from the actual time of development

^b^Actuator DOF = number of individually controlled actuators (zero means fully passive)

^c^Wrist can be assisted, resisted, limited or locked [29] in pronation/supination (PS), flexion/extension (FE) and radial/ulnar deviation (RUD)

**Table 5:** Overview of included dynamic hand orthoses classified as EVA glove

Name/ID	Country	Year range^a^	ISO abbr.	Reported function	Actuator DOF^b^	Wrist support^b^
Shields [111]	USA	1997	HdO	power assistance	3	Limited (FE, RUD)
SkilMate [56, 336]	Japan	2001–2004	HdO	power assistance	3	N/A
Matheson 1 [22, 23]	Australia	2011–2012	WHFO	general assistance	1	Limited (PS, FE, RUD)
Matheson 2 [22, 23]	Australia	2011–2012	FO	general assistance	2	Limited (PS, FE, RUD)

^a^Year ranges are determined by the year span between found literature sources and my differ from the actual time of development

^b^Actuator DOF = number of individually controlled actuators (zero means fully passive)

^c^Wrist can be assisted, resisted, limited or locked [29] in pronation/supination (PS), flexion/extension (FE) and radial/ulnar deviation (RUD)

**Table 6:** Overview of included dynamic hand orthoses classified as haptic device

Name/ID	Country	Year range^a^	ISO abbr.	Reported function	Actuator DOF^b^	Wrist support^b^
SKK Hand Master [68, 337]	South Korea	1999–2000	HdO	VR feedback	7	N/A
Koyama [338]	Japan	2002	HdO	VR feedback, teleoperation	0	N/A
Rutgers Master-II-ND [70]	USA	2002	HdO	VR feedback	4	N/A
LRP hand master [339]	France	2003	HdO	VR feedback	14	N/A
Stergiopoulos [65]	France	2003	HdO	VR feedback	2	N/A
Lelieveld [88, 340]	Japan	2006	FO	VR feedback	4	N/A
Nakagawara [341, 342]	Japan	2005–2007	HdO	tele-operation	6	N/A
Ryu [69]	South Korea	2008	WHFO	VR feedback	3	Not clear
CyperGrasp [343]	USA	2009	HdO	VR feedback	5	N/A
Fang [344, 345]	China	2009	HdO	teleoperation	5	N/A
Charoenseang [346]	Thailand	2011	HdO	VR feedback	9	N/A
Fontana [347, 348]	Italy	2009–2013	HdO	VR feedback, teleoperation	6	N/A
Dexmo F2 [349, 350]	China	2014	HdO	VR feedback	5	N/A
SPIDAR-10 [351]	Japan	2014	WHFO	VR feedback	20 (+1 wrist)	Assisted (PS) Limited (FE, RUD)
Jo [352, 353]	South Korea	2013–2015	HdO	VR feedback	5	N/A
SAFE Glove [354, 355]	USA	2015	HdO	VR feedback	6	N/A

^a^Year ranges are determined by the year span between found literature sources and my differ from the actual time of development

^b^Actuator DOF = number of individually controlled actuators (zero means fully passive)

^c^Wrist can be assisted, resisted, limited or locked [29] in pronation/supination (PS), flexion/extension (FE) and radial/ulnar deviation (RUD)

The majority of devices were home rehabilitation tools (56), followed by daily assistive tools (46), clinical tools (34) and research tools (9). Additionally, 16 haptic devices and 4 EVA glove mechanisms were found.

The resulting framework is split up into three figures, which are shown in Figs. 4, 5 and 6. The number of occurrences are added at the ends of the branches and grouped by year ranges.

## Discussion

### General

Results show that the development of dynamic hand orthoses has accelerated, as more than half of the found devices has undergone development in the last five years. Moreover, the amount of home rehabilitation and daily assistive tools indicate that the majority focuses on the development of devices that are used in a domestic setting, concentrating on being able to perform physical therapy at home or to help with ADL. Such observations can be linked to the trend where patient care is brought to their homes and workload on caregivers reduced [30–32].

The list of devices as presented in the tables, reveals several trends not covered in the framework. Only in rare cases, pathologies like tetraplegia, tendon injuries, arthritis or muscular weaknesses are specifically addressed in found literature. Consequently, these less targeted patient groups may fall short in specialized devices compared to more prevalent groups like stroke survivors. The tables also show that the wrist is often supported, albeit locked or assisted. In some cases, it is because the size of the mechanism or actuator module simply extends over the wrist. In other cases, however, the wrist is considered to be a crucial element in supporting overall hand function. Especially in the case of synergies or muscular weakness, supporting the combination of wrist and grasping function can be essential.

The presented framework illustrates the large span and variety of the solution space. The emerged collection of solutions can help future developers to form morphological overviews, to contemplate on the many possible combinations and to make concept choices. The unbalanced distribution and presence of outliers (i.e. very high or low number of occasions) indicate that some solutions are clearly more popular than others. A few are also never used (i.e. zero occurrences), such solutions were found by means of the framework or by inspiration from other fields of research (e.g. cineplasty from prosthetics, [33, 34]). It should be clear, however, that these numbers do not necessarily correlate to the functionality of the component. The reasons behind these differences remain speculations, but they can be due to performance, accessibility, popularity or because a solution is still experimental. Further detailed observations on the functionalities are described below per domain.

### Signal

#### Controller

Similar to the detailed review on training modalities [3], the passive-mirrored, corrective and path guidance modalities are used the least. They are also the least similar to the type of therapy a physical therapist can provide, and their low use implies that these methods are still in experimental phase. Little can be said about their efficacy, as the exact working principles behind a successful rehabilitation program are not yet fully known [17]. Nonetheless, their development helps in understanding these principles and exploring the full potential of involving robotic technology.

In general, the training modalities which are mostly used in dynamic hand orthoses, have the human in full authority over the movement. Due to the large amount of daily assistive tools, home rehabilitation tools and the inclusion of haptic devices, the assistive, resistive and passive training modalities show the highest frequencies and skew the distribution compared to a previous review on training modalities [3]. Especially for daily assistive tools, emphasis is more often put on regaining hand function rather than recovery of the physiological abilities. In these cases, assistive and self-triggered passive modalities are more popular.

#### Command signal

Detecting the user’s intention to serve as a command signal for the device is one of the larger challenges, because the control of the device is expected to be both intuitive and robust [35]. From the inspected dynamic hand orthoses, most state that measuring the command signal in series with the intended movement is most intuitive [23, 36]. This is also reflected in the results, as 100 cases use methods in series against 32 cases in parallel. The use of interaction forces and motions from the human plant is the most popular method of using a command signal in series. Here, issues due to sweat, sensor placement and signal quality are less interfering as compared to alternatives. Electromyography (EMG) as a measure of muscle activation is also often used and widely accepted in externally powered upper limb prosthetics, but more challenges are encountered in electrode placement and separation of signals [37–41]. Nonetheless, recent studies have shown that both methods (plant forces/motions and EMG) are feasible as a control interface [42, 43].

Parallel methods are considered less complex, useful for self-controlled mirror therapy [44, 45], or sometimes inevitable due to the absence of physiological signals directly relating to the intended motion [36, 46]. However, these methods can also take away useful functionalities (e.g. bimanual tasks, muscle use) and providing intuitive control is important to achieve user acceptance, stressing the advantages of using command signals in series whenever this fits within the design constraints.

Other methods that were encountered appear less feasible, less successful or experimental. For example, peripheral nerve interfaces (PNI) are not encountered as they can be considered as too invasive; measuring brain activity through electroencephalography (EEG) has an increased risk of false positives and negatives (even with a binary system [47]); force myography (FMG) remains in experimental phase [7]; and, mechanomyography (MMG) is subject to environmental sounds and limb-movement artifacts [7].

#### User feedback

A large portion of the investigated devices (67 out of 165) use augmented user feedback. Especially multimodal feedback is a popular method of providing the user with additional cues. Here, VR environments are often used as a platform to provide audiovisual cues (e.g. [48]), audiovisuohaptic cues (e.g. [49]) or haptic rendering (e.g. [50]). Amongst others, this can enhance a sense of reality or provide information on performance. Augmenting unimodal feedback (i.e. visual, auditory or haptic) can also be used in various manners. For example, the force exerted by the device can be visualized [51], music can facilitate motor output [52] and stimulation of the muscle spindles through vibrations can give an enhanced sensation of motion to further enhance rehabilitation success [53, 54].

From a different perspective, augmented feedback can be used to compensate for an attenuation of haptic feedback [55, 56]. A spatial separation between the palmar surface and the environment can affect force perception [57], hence facilitating tactile sensation is considered to be of great importance in dynamic hand orthoses [58].

The design of augmented feedback signals, however, should be considered carefully. It does not always work effectively [55] and may even prove to be counterproductive [59]. Determining the ideal form of augmented feedback signals is challenging, hard to verify and in many cases related to task complexity [24]. Nonetheless, proper designs have shown potential in robot-aided rehabilitation [60].

### Energy

#### Energy storage

The usage of components for energy storage is rarely reported, which is reflected by the low number of cases where this could be determined (73 out of 165). Of these cases, the method of energy storage is usually a consequence of choices in actuation, which is why electric batteries are often used because of the high use of electric/magnetic actuators. It should be noted, however, that tapping energy from a centralized system (e.g. mains electricity or compressed air systems) was not considered. It’s usage is in many cases hard to verify from available literature and its effects on portability are covered in the mechanical domain.

The most-often used form of energy storage is with elastic energy, of which a helical spring is the most straightforward example. They are often added to realize antagonistic movement when the primary actuation or transmission method is not able to do so [61]. Other usages include applications where unidirectional and passive forces are sufficient to overcome an impairment, which is the case when compensating hyperflexion [62]. A special case of utilizing elastic energy lies in compliant structures. Aside from introducing mechanical potential energy, they can function like a mechanism and provide for an articulating and load-bearing structure. Compliant mechanisms are both efficient and inherently flexible [63], but also introduce complications in defining rotation centers and there is a careful balance between stiffness and elasticity [23].

#### Actuation

The most prominent result from the trends on actuation components is the large amount (113 out of 165) of devices that use a form of electromagnetic actuation. DC motors have the upper hand within this group, but reasonings behind this choice are hard to retrieve. Reported arguments include the increased possibilities for both position and torque control [64], high mechanical bandwidth [65] and general performance in the torque-velocity space [13]. Such properties appear most useful in applications where variability in control strategies is sought-after or when high-frequent perturbations or interactions need to be applied. For applications that focus more on general assistance, the lower torque-to-speed ratios of DC motors need to be reduced to coincide with the higher ratio demands for human movement. As a result, gearheads are added to reduce the high speeds, adding backlash and reducing inherent backdrivability of the device [65]. An interesting development here lies in the twisted string actuation system, which reaches high reduction ratios by twisting strands on one end and creating linear motion on the other end [66, 67]. Alternatively, ceramic piezoelectricity as used in ultrasonic motors can also provide for a more suitable torque-to-speed ratio. They are silent, have high power-to-weight ratio and are able to facilitate free motion [68]. However, they also require high voltages [69] and show hysteresis [68].

An often mentioned substitute for electromagnetic actuation, is the use of pneumatic actuation. They are intrinsically compliant, lightweight, act similar to natural muscles and high power-to-weight ratios are reported [23, 58, 61, 70–74]. Still, no commercial pneumatically powered prosthesis or orthosis exists to date to our knowledge. The main reported drawbacks are difficulties in control, expensive components and low bandwidth [69, 71, 75]. The stated arguments, however, impress as ambiguous due to vague definitions and lack of comparison with design requirements. For example, definitions for power-to-weight ratios are often unclear [76] and a distinction can be made between high- and low-force bandwidth [77]. Concerning the latter, human force control operates at around 7 Hz [78] and rehabilitation does not necessarily require high bandwidths [4], displaying values that do appear within range of pneumatic actuators.

Other methods of actuation appear to be more experimental or impractical. The natural muscle can be used as an actuator and is the crux in body-powered prosthetics. Although applicable for local impairments at the hand, this becomes less practical in orthotics when the muscle itself requires support, as this would add the need for yet another force amplifier. Active polymers appear more promising, being thin, lightweight, compliant and able to perform both sensing and actuation. However, in [79], it was stated that fundamental enhancements would be required for feasible use in upper limb prosthetics. Similar to shape memory alloys [80–82], forces are generally low and take time to build up (i.e. low bandwidths), which results in the need for large stacked configurations [83, 84].

### Mechanical

#### Transmission

No existing studies were found that presented a form of categorization on transmission components usable for dynamic hand orthoses. Consequently, the results and interpretation are based on (and limited by) a categorization from the authors’ perspective. Some approaches can be considered as a direct consequence from design choices in the energy domain. For example, gears are most often used to alter DC motor speeds and compliant mechanisms integrate both energy storage and transmission. Other approaches are more a result of choice in mechanism, where *n*-bar linkages are well-known methods of facilitating path trajectories. Nonetheless, additional notable approaches can be reviewed and coupled with reported argumentations.

The most arguments are reported for pulley-cable and Bowden cable systems. Pulley-cable systems are spatially constrained and require a continuous control of cable tension to maintain traction on the pulleys [13, 85, 86]. Bowden cable systems, on the other hand, are a type of cable-conduit and are essentially flexible, but introduce variable and high friction forces dependent on curvature [87–91]. Nonetheless, both cable systems most resemble the tendon mechanism in the natural hand [61, 92–97] and are often an effective method of proximally placing the actuators to reduce the inertia of moving parts [13, 85, 96, 98, 99].

Fluidic transmissions are generally more efficient for larger channel diameters, which could explain the low use in dynamic hand orthoses (3 out of 165). Despite this, hydraulic transmissions remain promising at similar scales [100] and are able to provide a more efficient alternative compared to a similar cable mechanism [101]. In comparison with hydraulics, a pneumatic transmission can offer faster responses due to the use of low-viscosity fluids [69, 100], but is not encountered in the included dynamic hand orthoses.

#### Mechanism

The alignment of anatomical and mechanical joints is the essence of many mechanical design papers on hand orthoses, which is especially the case for exoskeleton-based devices [4]. Misalignments may cause numerous sources of discomfort to the user, resulting in possible frustration by the user, rejection of the device and eventual hindrance in the rehabilitation program [102]. Even tissue damage can occur, where pressure sores, joint dislocations or cartilage damage are among the possibilities depending on the user [102, 103]. The main design challenges lie in limited available space, differences in hand sizes and coping with the compliance of skin tissue. Additionally, the rotation axis of a finger joint is not constant [104], i.e. polycentric. Despite the latter, however, almost half of the dynamic hand orthoses use monocentric rotation (79 out of 165). This includes the more straightforward hinge joints [61], but also those that use a virtual center of rotation with fixed rotation axis (e.g. concentric rotation in [105]). In these cases, the rotation centers need to be manually aligned and results in a time-consuming process for different hand sizes [90]. This is where self-aligning joint centers are often-used alternatives. They are able to adapt to various hand sizes [44] and prevent strong discomfort for the user [96, 98]. Self-aligning mechanisms are essentially polycentric and conform to whatever rotation the anatomical joint imposes. Moreover, efficiency is increased as the device finds less resistance from the user.

End-effector-based devices omit the constriction of joint movement by only moving the most distal end of the fingers [4], forming a kinematic chain with the ground. This makes it advantageous over exoskeleton-based devices [106], but also less suitable for applications with more strict design constraints on portability (i.e. home rehabilitation and daily assistive tools).

A general trend towards simplification of the hand kinematics can be seen. This includes the introduction of couplings by force (i.e. underactuation) and by motion (i.e. constraints) in order to reduce the complexity of the device. These methods are similar to the mechanical couplings and control synergies that exist in the natural hand [104, 107]. This concept can be generalized under the term functional degrees of freedom (fDOF) [108], which means that complex movement patterns can be generalized and achieved by less complex actuation strategies. This is a viable approach for dynamic hand orthoses as complex multi-DOF movements are unnecessary for many rehabilitation purposes [60, 109] and grasping patterns that are used during ADL can be generalized [110]. Underactuation, in particular, is a popular method as it reduces weight and complexity [65, 74, 86, 93, 97, 109, 111] and it facilitates passive adaptation for better object manipulation [86, 94]. From the results, it appears that intrafinger (i.e. across joints) underactuation is preferred, as opposed to interfinger (i.e. across fingers) underactuation which is an upcoming feature and allows passive adaptation to 3D objects [93, 112].

## Conclusion

A high quantity of dynamic hand orthoses was gathered and shows that their development is becoming increasingly prevalent. A framework was developed in an attempt to collectively analyze the diverse solution space, whose general methodology can be used for other mechatronic systems that interact with the human. The investigated solution space reveals several outliers, for example the preference for EMG or force/motion control and electromagnetic actuation. There are also less-used solutions that do seem feasible, like compliant mechanisms, fluidic transmission/actuation and interfinger underactuation. By no means is the framework complete, as more branches can be added to the tree diagrams that expand and extend further into the solution space at increased level of detail. Even so, a comprehensive analysis was performed that can be used as a general exploration on mechatronic design of dynamic hand orthotics—and possibly other related fields as well.

## Abbreviations

DMD, duchenne musculuar dystrophy; ADL, activities of daily living; CPM, continuous passive motion; VR, virtual reality; EVA, extra-vehicular activity; DOF, degree of freedom; fDOF, functional degrees of freedom; EEG, electroencephalography; PNI, peripheral nerve interface; EMG, electromyography; MMG, mechanomyography; FMG, force myography

## Additional file

Additional file 1 An Excel spreadsheet which contains obtained information from all devices and categorization into the presented framework. (XLSX 143 kb)
